# Harnessing retinal phagocytes to combat pathological neovascularization in ischemic retinopathies?

**DOI:** 10.1007/s00424-022-02695-7

**Published:** 2022-05-07

**Authors:** Anne Klotzsche-von Ameln, David Sprott

**Affiliations:** Institute of Physiology, Faculty of Medicine, Technische Universität Dresden, Dresden, Germany

**Keywords:** Retinopathy, Pathological angiogenesis, Inflammation, Mononuclear phagocytes, Microglia, Macrophages

## Abstract

Ischemic retinopathies (IR) are vision-threatening diseases that affect a substantial amount of people across all age groups worldwide. The current treatment options of photocoagulation and anti-VEGF therapy have side effects and are occasionally unable to prevent disease progression. It is therefore worthwhile to consider other molecular targets for the development of novel treatment strategies that could be safer and more efficient. During the manifestation of IR, the retina, normally an immune privileged tissue, encounters enhanced levels of cellular stress and inflammation that attract mononuclear phagocytes (MPs) from the blood stream and activate resident MPs (microglia). Activated MPs have a multitude of effects within the retinal tissue and have the potential to both counter and exacerbate the harmful tissue microenvironment. The present review discusses the current knowledge about the role of inflammation and activated retinal MPs in the major IRs: retinopathy of prematurity and diabetic retinopathy. We focus particularly on MPs and their secreted factors and cell–cell-based interactions between MPs and endothelial cells. We conclude that activated MPs play a major role in the manifestation and progression of IRs and could therefore become a promising new target for novel pharmacological intervention strategies in these diseases.

## Ischemic retinopathies

Retinopathy of prematurity (ROP) and diabetic retinopathy (DR) are the most important forms of IR. They cause a significant loss of life quality and productivity and are among the leading causes for blindness in their respective age groups. Development of IRs is complex and involves several neuronal, vascular, and inflammatory processes. Both ROP and DR progress in two main phases, an early degenerative phase (termed “non-proliferative” phase in DR and “vasoobliterative” phase in ROP) that is followed by a second “proliferative” phase.

The early phase is characterized by capillary dropout in DR and by growth cessation of vessels in ROP, respectively. In both pathologies, the vascular abnormalities then result in retinal ischemia and hypoxia that can evoke a second over-compensatory, proliferative phase that is characterized by dysregulated/pathological angiogenesis (= neovascularization). The pathological retinal neovessels (“tufts”) created in this way are ectopic and can invade the vitreous cavity, which is associated with increasing risk for retinal detachment. Additionally, since the ectopic vessel structures are frequently malformed, fragile, and leaky, this can lead to accumulation of plasma proteins and cell debris in the retina that further manifest in vitreal hemorrhages and vision loss [1, 36, 39, 70, 102].

### Retinopathy of prematurity

ROP is the leading cause of childhood blindness worldwide, but only occurs in prematurely borne infants (in humans defined as birth before 37 weeks of gestation). However, as survival rates of premature children with a very low gestational age rise in many areas of the world, the numbers of ROP cases increase [61, 70].

The formation of retinal vasculature is initiated by the beginning of the fourth month of gestational age. At this time point, blood vessels start to sprout radially from the optic nerve towards the *ora serrata*. The process of retinal vasculature establishment is complex, requires the interactions between different cell types, and, in humans, is completed only shortly before full-term birth under normal circumstances [39, 70]. However, in preterm infants, retinal vessel development is incomplete and therefore ROP affects mainly the retinal vasculature.

**Fig. 1:**
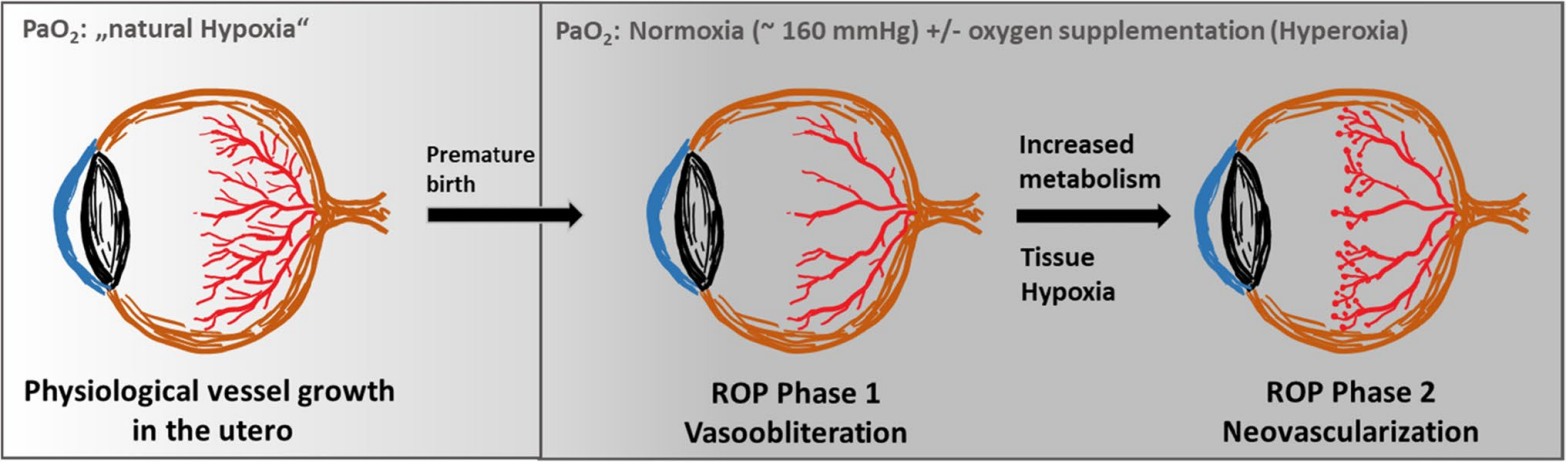
Schematic illustration of ROP disease manifestation. Physiological retinal vessel growth in humans starts at the beginning of the fourth month of gestational age (“natural hypoxia”) and is normally finished shortly before full-term birth. Accordingly, premature infants have incompletely vascularized retinas. After birth, loss of nutrients and physiological growth factors provided at the maternal–fetal interface together with the increased oxygen pressure (oxygen pressure of ambient air ~ 160 mmHg; plus additional oxygen supplementation) result in a persistently undervascularized (vasoobliterative phase) and later hypoxic retina. This causes excess production of VEGF and other oxygen-regulated vascular growth factors, resulting in pathological retinal neovascularization (neovascularization phase)

One of the main determinants for physiological vessel development is a tightly regulated oxygen pressure in the angiogenic tissue. The normal partial oxygen pressure (PaO_2_) in utero is approximately 50 mmHg by the end of pregnancy [70]. By comparison, ambient room air has a much higher oxygen pressure of around 160 mmHg PaO_2_. Furthermore, many prematurely born infants receive additional oxygen supplementation to allow their survival ex utero. Thus, retinas of newborns receiving oxygen supplementation are exposed to a dramatically higher oxygen pressure (hyperoxia) compared to the situation in utero (Fig. 1) [70].

Oxygen excess suppresses vessel growth in the retina and causes constriction of already-developed vessels. As a result, avascular hypoxic areas can form within the retina during this vasoobliterative phase of ROP [70, 94]. Following this phase, metabolism and oxygen consumption of the growing undervascularized retinal tissue increases, but adequate supply is not available due to the lack of functional vasculature. This leads to the development of tissue hypoxia and thereby to a pronounced upregulation of hypoxia-regulated angiogenic growth factors, such as the vascular endothelial growth factor (VEGF) [39, 94].

During the subsequent proliferative phase of ROP, the disproportionally high levels of VEGF cause the outgrowth of pathological vessel structures (tufts), which grow from the retina into the vitreous. These neovascular tufts are dysfunctional and leaky due to an impaired blood-retina barrier (BRB) (Fig. 1) [70]. Ultimately, in most infants, pathological tufts are spontaneously cleared and ROP resolves by itself, resulting in a normalization of the retinal vessels [23, 41, 48]. However, in a substantial amount of cases, fibrous scar tissue remains, which tends to cause traction on the retina, and can, in the worst case, lead to retinal detachment and possibly blindness [70]. In addition, ROP often decreases visual acuity even years after resolution and increases the risk of developing myopia [13].

### Diabetic retinopathy

Diabetes is a complex metabolic disorder associated with hyperglycemia, hypo-/hyper-insulinemia, dyslipidemia, and hypertension. The systemic metabolic and cardiovascular alterations in diabetic patients affect all retinal cell types and will gradually, with a high likelihood, cause the development of DR and vision impairments/blindness in the majority of patients. DR is characterized by endothelial cell dysfunction, but other cell types such as microglia, Mueller cells, and neurons play crucial roles as well [2, 64]. Therefore, DR is mainly a microvascular disease that is linked to chronic inflammation and retinal neurodegeneration. However, the exact underlying molecular mechanisms, the chronological order, and the complex interplay between the different cell types of the retina are not fully understood at this point.

In the healthy retina, the microvasculature is a highly organized network of stable and tight microvessels that allows efficient nutrient supply and waste product removal in a tissue with high metabolic activity [64]. An important prerequisite for these properties is a sufficient pericyte coverage on the extraluminal site of the endothelium, which regulates endothelial cell survival and supports the integrity of the BRB that protects the retina from potentially harmful substances in the blood (including immune cells) [88].

Long-term diabetes, with chronic hyperglycemia, induces damage to both endothelial cells and pericytes, leading to pericyte detachment and migration, as well as apoptosis, resulting in a dysfunctional BRB [36, 102]. In addition, diabetes-related cell stress causes the endothelium to produce excessive amounts of vascular basement membrane components, leading to overall basement membrane thickening and formation of structural and functional lesions [83, 96]. Pericyte loss and thickening of vascular basement membrane sheets are the first characteristic alterations of DR.

**Fig. 2:**
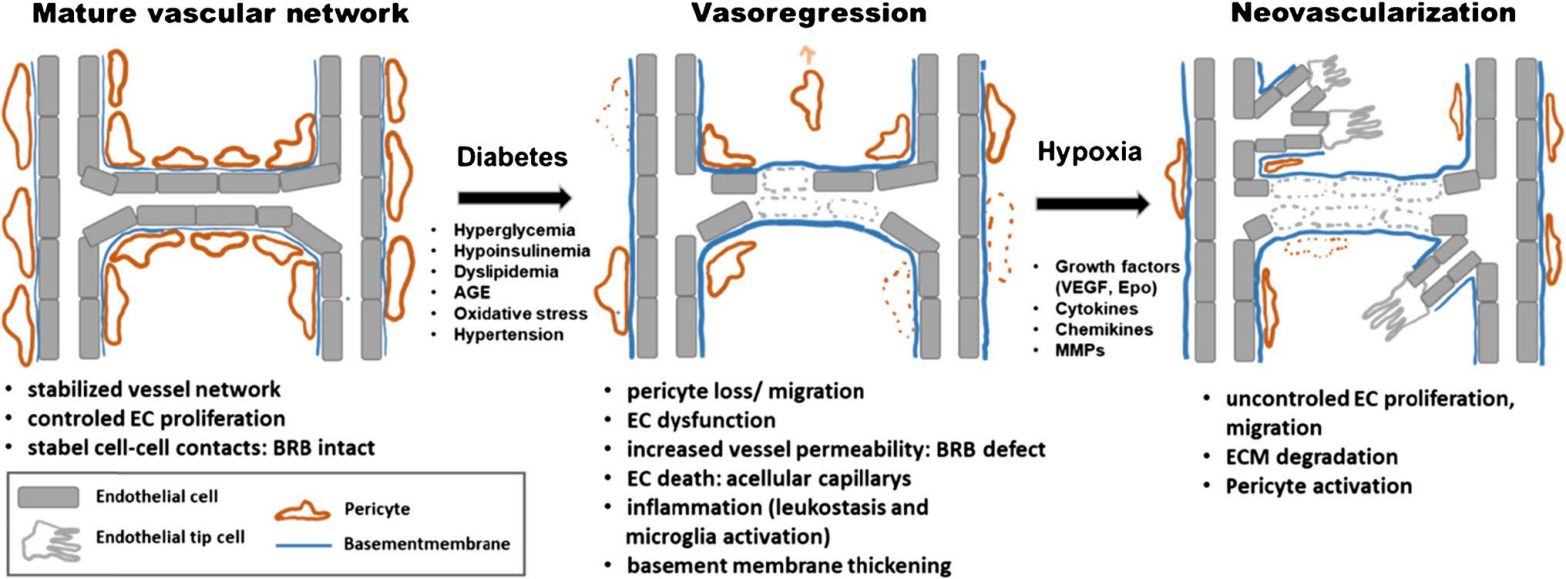
Summary of the main pathogenic events causing pathological neovascularization in the retina of patients with DR. In healthy retinal capillaries, adequate pericyte coverage supports endothelial cell survival and integrity of blood retina barrier. Long-term diabetes induces alterations and damage in several cell types resulting in progressive vasoregression. Occluded remnants of capillaries are no longer perfused, leading to tissue hypoxia and a subsequent upregulation of survival/growth factors such as VEGF. As a consequence, ischemia/hypoxia-induced, pathological neovascularization is triggered. EC endothelial cells, BRB blood retina barrier, ECM extracellular matrix, AGE advanced glycosylation end products, VEGF vascular endothelial growth factor, Epo erythropoietin

Subsequently, stressed endothelial cells upregulate adhesion molecules such as intercellular cell adhesion molecule (ICAM)-1 and vascular cell adhesion molecule (VCAM)-1, causing inflammatory processes such as enhanced leukocyte adhesion and transmigration in the microvessels [1, 102]. These phenomena further enhance the loss of endothelial cells, promote vascular permeability, and ultimately cause the breakdown of the BRB. As a consequence, capillaries degenerate and leave behind non-perfused capillary residues (acellular capillaries), which display the first relevant morphological signs observed in patients with DR [36]. In parallel, due to the metabolic changes and as a consequence of the reduced retinal blood flow, dysfunction and degeneration of retinal neurons and glial cells may occur [1, 97]. These processes can cause gradual thinning of the nerve fiber layer and progressive loss of vision (Fig. 2) [1, 53, 97, 102]. If left untreated, the non-proliferative phase of DR can progress further into a proliferative phase (proliferative diabetic retinopathy (PDR)) that is defined by pathological neovascularization. In response to the progressive retinal capillary dropout, the retinal tissue becomes first ischemic and hypoxic, which, like in ROP, results in the activation/stabilization of the transcription factors of the hypoxia-inducible factor (Hif) family in several cell types, e.g., endothelial and glial cells. Consecutively, hypoxia-induced growth factors (e.g., VEGF, angiopoietins, and erythropoietin (Epo)) and pro-inflammatory cytokines (such as TNFα, IL-6, and IL-1β) are released and promote the formation of new, yet fragile, aberrant and leaky vessels, resulting in vitreous hemorrhage, which can cause retinal scars and detachment, potentially leading to irreversible vision loss and total blindness (Fig. 2) [1, 36, 102].

Moreover, formation of diabetic macular edema may occur at any stage of DR (in both the non-proliferative and the proliferative phases), which is a result of BRB breakdown and elevated vascular permeability causing fluid accumulation (swelling) within the retina. This enhances cell death and may result in further visual impairment and/or loss [1].

### Current therapies for ischemic retinopathies

Ischemic retinopathies are treated with therapeutic strategies such as retinal coagulation, vitreoretinal surgery, and intravitreal injections of anti-VEGF agents.

Ablation of the avascular retina through coagulation treatment was one of the first routinely performed treatment options and can be achieved either by the use of laser light (laser coagulation) or via local tissue freezing (cryocoagulation). This method aims to destroy peripheral retinal areas in order to reduce the metabolically active tissue, total oxygen consumption, and pro-angiogenic factor load, which ameliorates exuberant angiogenesis and prevents further complications such as retinal detachment [70]. There are however drawbacks of this method, as coagulation treatments can permanently reduce the field of vision. Moreover, in ROP patients, retinal coagulation treatment can increase the risk for developing myopia [12, 94].

As an alternative to coagulation therapies, especially in ROP, anti-VEGF therapy allows easier administration under topical anesthesia and preserves the peripheral retina, which avoids the risk of visual field defects associated with photocoagulation [94]. The ability of VEGF to promote both vascular permeability and angiogenesis made it an attractive target to battle vascular dysfunctions observed in both severe DR and ROP. Intravitreal injections of anti-VEGF agents have led to significant progresses in the treatment of patients with IR and have become the primary treatment option for ROP in many countries. In spite of the significant success, anti-VEGF therapy has a number of limitations. For instance, intravitreally injected anti-VEGF can enter the blood circulation and can thereby detectably lower systemic levels of VEGF [37, 121]. In infants with ROP, this may have severe effects on developing organs, including the lung, brain, and kidney [39]. Potential systemic adverse effects of anti-VEGF treatments in patients with DR include thromboembolic events, hypertension (systemic and ocular), gastrointestinal disorders, and kidney disease [31, 87, 90, 111]. Furthermore, repeated long-term intraocular injections may provoke intraocular inflammation and infectious endophthalmitis [31]. In addition, anti-VEGF treatment is mostly demonstrating its efficacy in the late phases of DR, and only approximately half of the patients respond fully to the treatment [97, 104].

In summary, current treatment options for IR can counter pathological retina angiogenesis, but occasionally exhibit side effects and cannot reliably prevent blindness in all cases. Therefore, new methods need to be developed. These should ideally not be based on the targeting of a trophic factor like VEGF that is necessary for a wide array of physiological processes, but should rather strive to counter early triggers of pathological neovascularization and shift it towards healthy revascularization. Another drawback of the current treatment options is that they are not preventive, as they are not able to counter tissue stress and destruction during the earlier degenerative phases of IRs, but instead merely aim to limit the manifestation of the second proliferative phase.

Thus, in order to develop improved treatments, further studies and a better understanding of causative factors triggering pathological angiogenesis will be necessary. One such factor could be retinal inflammatory cell activation, since there are many indications that inflammation is implicated in provoking, promoting and resolving pathological angiogenesis. However, the complex interconnection of IR with inflammatory factors and the immune system is not well understood to date and will require further elucidation.

### Use of the mouse “oxygen-induced retinopathy” model to study ischemic retinopathies

Due to ethical restrictions and the limited availability of human samples from patients with IR or even healthy tissue for proper controls, animal models are necessary to understand the underlying mechanisms for the development of the pathologies.

**Fig. 3:**
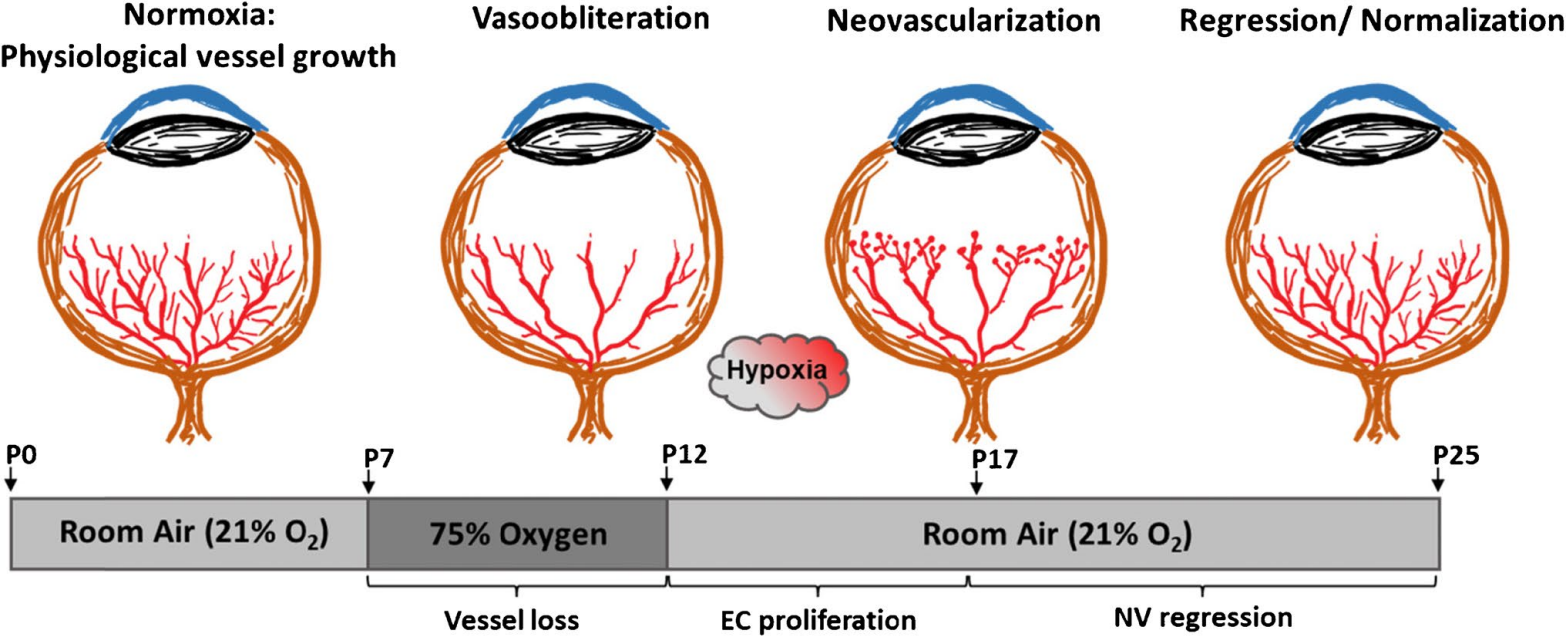
Schematic illustration of the mouse OIR model. Neonatal mice are kept in ambient air (21% oxygen) from birth until postnatal day 7 (P7); meanwhile, normal vascular development starts. At P7, mice are then exposed to 75% oxygen, resulting in the inhibition and regression of retinal vessel growth (vaso-obliteration). At P12, mice are returned to ambient air. The drop in oxygen pressure leads to the development of hypoxia in avascular retinal areas, triggering both normal vessel regrowth and pathological neovascularization. Pathological vessel growth reaches its maximum at P17 and spontaneously regresses thereafter until the retinal vasculature is completely normalized by P25

The primary animal model to study pathological neovascularization in the retina is the mouse model of oxygen-induced retinopathy (OIR) [105]. In this model, neonatal mice (pubs) are subjected to hyperoxia (75% oxygen) from postnatal day 7 (P7) until P12, recapitulating the shifting oxygen levels for prematurely born human infants. Similar to the situation in human counterparts, this treatment firstly inhibits retinal vessel growth and secondly causes a significant regression of the retinal vasculature that already developed until that point. This so-called vaso-obliteration mimics the first phase of human ROP, and its extent can be determined by measuring the avascular area in retinal whole mounts. On P12 in the OIR model, the mice are then returned to ambient air oxygen levels, causing hypoxia manifestation in the avascular areas of the retina [23, 101]. This hypoxic stimulus boosts the expression of angiogenic factors (such as VEGF) that trigger both physiological vessel re-growth into the avascular area as well as the formation of the pathological tufts. The neovascularization phase, which reaches its maximum at P17 in mice, is similar to the second phase of human ROP and furthermore mimics many aspects of the proliferative phase of human DR. Also similar with human ROP is the spontaneous regression of pathological vessels, which occurs between P17 and P25 in mouse OIR (Fig. 3) [23].

However, in spite of its many advantages, the mouse OIR model, as all experimental models, has limitations and recreates only some aspects of human IR. To begin with, patients suffering from ROP generate avascular areas in the peripheral retina, while mice exposed to high oxygen treatment develop ischemia and vaso-obliteration in the center of the retina [38]. It is also worth mentioning that rodents experience much higher arterial oxygen levels when given similar inspired oxygen levels as premature infants and that neonatologists generally avoid high oxygen levels in the perinatal period, while the OIR model uses high oxygen [39]. Moreover, the vascular development in mice always starts after birth, whereas even prematurely born human infants already show some degree of retinal vascularization at this time [45, 98]. For this reason, the mouse OIR model is required to use healthy newborns instead of prematurely born animals. But physiological vessel development is adapted to different oxygen levels in mice and humans, and therefore, this method cannot fully compensate the inherent differences in vessel development between the two settings, especially since non-preterm animals do not exhibit the comorbidities of human preterm infants [39]. Taken together, these differences between human ROP and the mouse OIR model create a lack of comparability that should be kept in mind when interpreting results obtained from this animal model in human context.

When using the OIR model to simulate aspects of proliferative DR, the limitations of this model have to be considered especially well. OIR pubs will naturally not exhibit the systemic hyperglycemia and the resulting metabolic (e.g., chronic hyperglycemia) and cardiovascular alterations that are normally present in diabetic patients. Therefore, the model cannot be used to answer questions about the causation of DR or about. However, as rodents exposed to experimental diabetes models do not develop the proliferative stages of DR, there are unfortunately few alternatives to the OIR model to date for studying proliferative retinopathy in this context.

Despite the aforementioned shortcomings, the mouse OIR model has many similarities with human IR and, with the use of transgenic mice, makes it possible to study cell type-specific functions of individual genes on the development of the disease [23, 39, 101]. In this regard, several signalling pathways and the individual function of different cell types, as well as cell–cell interactions, which are important to better understand human IR have been identified using the mouse OIR model.

### Retinal cellular stress during ischemic retinopathies triggers local tissue inflammation

Even under physiological conditions, the retina possesses a high oxygen consumption rate and constant cellular turnover. These special features of the retina create a demand for efficient blood supply and recycling processes to allow its functionality and to keep its highly ordered structure stable. Under normal conditions, this is ensured by the dense network of micro vessels and several specialized supporting cell types, all of which are necessary for retinal health. As stated before, the retinal microvasculature becomes compromised during IR, impairing the supply chain for retinal tissues und thereby causing deprivation of oxygen (hypoxia) and nutrients. This creates a tissue microenvironment with profound oxidative, cellular, and endoplasmatic reticulum stress [9, 69], all of which have the potential to induce tissue apoptosis [25] and to further drive disease progression.

A hallmark of the cellular response to retinal hypoxia is the activation of the well-described Hif-pathway [95, 123]. Another adaptation mechanism to stressful microenvironments such as ischemia/hypoxia and oxidative stress that is utilized by many cell types is the activation of autophagy, a cellular recycling process that protects cells from apoptosis and re-initiates cellular homeostasis [91, 106, 116]. Consistently, mice exposed to the OIR model showed a marked activation of autophagy, especially in the vascular component. Autophagy activation in this context was observed before and during neovascularization, which suggests that nutrient deprivation and/or cellular stress take place in the retina throughout all pathological stages of this retinopathy model [106]. Similar to the situation in the OIR model, early activation of retinal autophagy was also observed in diabetic rats, supporting the notion that the onset of the cellular stress factors that necessitates autophagy activation may be a general phenomenon in retinopathies [84].

Cellular stress of retinal tissues during IR manifestation does not only lead to the activation of cell survival pathways, it moreover triggers inflammatory processes. Inflammation has the potential to both exacerbate IR development by starting a vicious cycle of causing further inflammatory activation, as well as to trigger a healing response that promotes resolution of the disease. A growing body of evidence supports the hypothesis that inflammation is a key modulator in the development and progression of IR. In DR, the participation of inflammatory processes is self-evident, as glucose at high levels boosts the formation of inflammation-inducing advanced glycation end products (AGEs) [5, 114]. Transiently increased AGE levels in long-term diabetics trigger the production of pro-inflammatory factors, such as TNFα, IL-1β, IL-8, and MCP-1 and thereby cause chronic low-grade systemic inflammation, also in the retina, and this mechanism is suspected to play a major role in the causation of DR [1, 97, 102, 128]. Consistently, in models of induced diabetes in rodents, the retina exhibits a pro-inflammatory profile [14]. Retinal inflammation may furthermore be causally related to the development of DR, since application of several anti-inflammatory compounds dampened retinal dysfunction in rodent models of diabetes [16, 17, 103]. Inflammation may also be an important modulator in the development of ROP. According to some works, elevated levels of inflammatory markers at prenatal and perinatal stages correlate positively with chances of ROP onset and may therefore be risk factors for this disease [19, 65]. Additionally, consistent results were obtained in animal models, where elevated levels of TNFα and IL-1β could be detected in rodents exposed to the OIR model [93]. Moreover, increased inflammation may be causally linked to the formation of pathological angiogenesis in these models, and reduction of inflammation through genetic depletion of TNFα or through administration of the anti-inflammatory food supplements ω-3-polyunsaturated fatty acids (PUFAs) was able to reduce neovascularization [22, 33]. Similarly, to these results seen in animal models, the anti-inflammatory steroid dexamethasone was able to reduce ROP occurrence in prematurely bone infants, further hinting at a potential link between inflammation and IR development [44].

### Emergence and source of activated retinal mononuclear phagocytes during ischemic retinopathies

Inflammatory processes in the retina do not only propagate local cell stress and apoptosis induction, but they also have the potential to activate and attract mononuclear phagocytes (MPs). Indeed, several works have shown that the number of MPs in the retina increases during the course of the OIR model and that these cells accumulate especially in areas of ischemia and neovascularization, which is suggestive of a possible role for MPs in IR [10, 24, 27, 51, 73, 74, 124]. In fact, some studies have identified retinal MPs as key modulators of neovascularization [73]. The surge in retinal MP numbers seen in OIR models and IR patients [3, 122] raised the question where these cells originate. At first glance, it seems unlikely that retinal MPs originate from blood-derived monocytes, since the retina is an immune privileged tissue, where the BRB prevents open communication between immune system and retinal tissue. For this reason, circulating macrophages and monocytes rarely penetrate the BRB to enter the retinal tissue under physiological conditions [109, 125]. However, when the retina experiences extensive stress conditions, such as in IRs, the BRB becomes pervious and monocyte-derived MPs from the bloodstream and other sources (e.g., ciliary body, optic nerve, vitreous, and choroid) become able to rapidly migrate to the site of retinal injury [21, 50, 51]. This makes blood derived monocytes a possible source for the surplus retinal MPs observed in IR models. However, while earlier studies indeed indicated a major influx of blood-derived MPs into the diseased retina [27, 51], more recent data suggest that blood-derived MPs may only play a minor role, whereas retina-resident MPs, the microglia, may represent the predominant MP population in areas of retinal ischemia and neovascularization [10]. This suggests that microglia are specifically attracted to pathological tufts and may play a leading role in the development of retinopathies. Of note, in the healthy adult retina, microglia are mainly located in the inner retinal layers, such as the ganglion cell and the inner and outer plexiform layers, while they are rarely found in the inner nuclear layer and are absent in the outer nuclear layer—the latter being the predominant location during neovascular tuft formation [1, 53, 82].

Under physiological conditions, microglia are multifunctional self-renewing, relatively long-lived, tissue-resting immune cells that constantly move through the tissue and screen with their extending and retracting long processes the retinal microenvironment to preserve tissue homeostasis [1, 53]. They support the function and development of other retinal cell types, including neurons, glial cells, and endothelial cells, by either direct interaction with them or indirectly via secretion of growth factors, cytokines, or neuroprotective and anti-inflammatory mediators. Through these mechanisms, microglia influence neurogenesis, axonal growth, and even the formation of blood vessels [1, 18, 67].

**Fig. 4:**
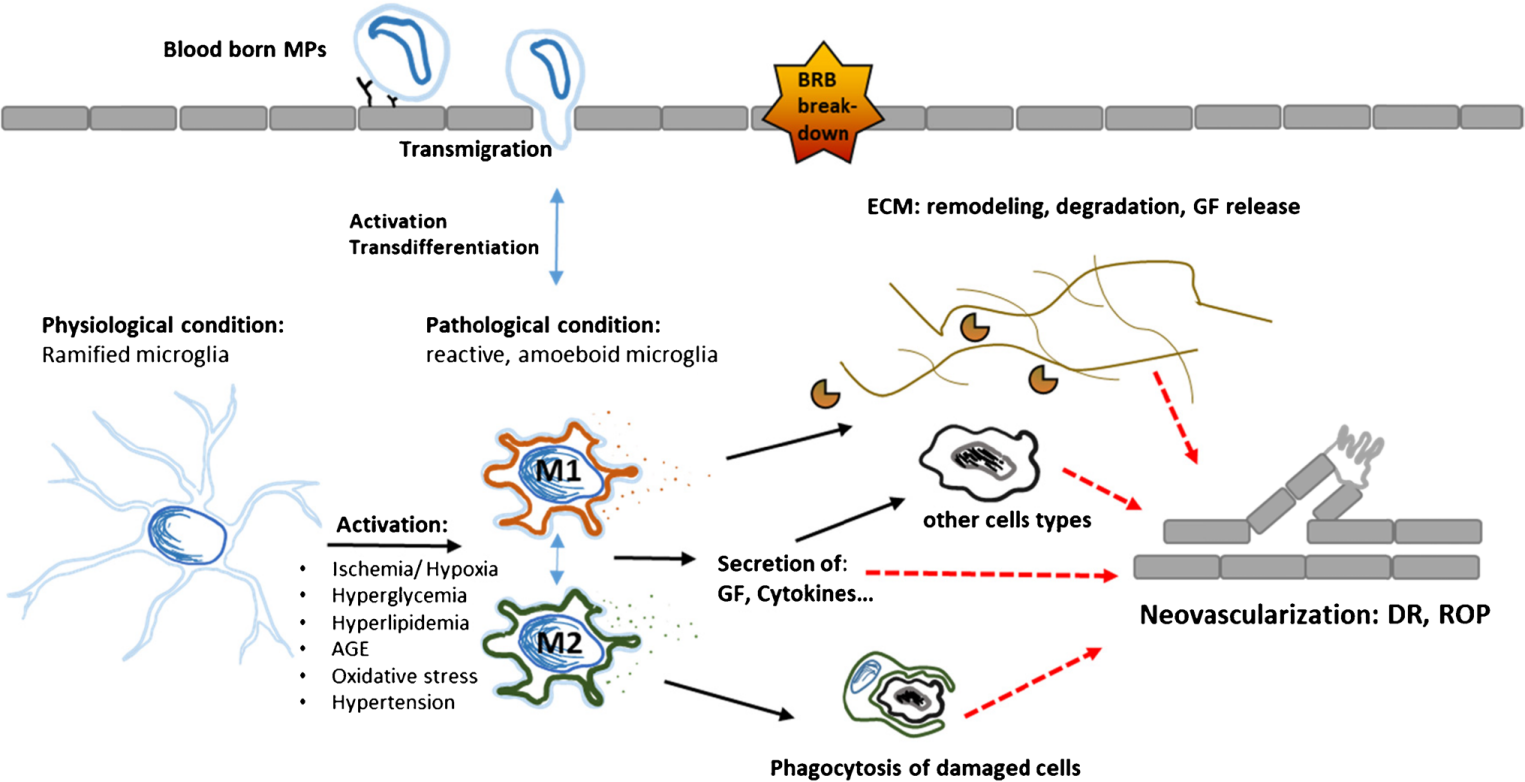
Summary of the action of MPs causing pathological neovascularization in the retina. Two immune cell populations are described to affect pathological neovascularization. These are either macrophages derived from the circulation or resident microglia. In the stressed retina, ramified microglia become activated and differentiate into an M1 or M2 phenotype, which goes along with respective changes in cell morphology, proliferation, migration, phagocytosis, and alterations in cytokine/growth factor/protease production. MP mononuclear phagocyte, EC endothelial cells, BRB blood retina barrier, ECM extracellular matrix, GF growth factor, AGE advanced glycosylation end products VEGF vascular endothelial growth factor, Epo erythropoietin

However, as the delicate retinal homeostasis is unbalanced during IR development and cell stress and inflammation manifest in the tissue, microglia detect these changes and become activated. Microglial activation has indeed been documented in clinical studies from patients with IR [3, 18, 122, 124], as well as in the mouse model of OIR [10, 32, 73]. The activation of microglia in the retina is a tightly regulated process that involves changes in their morphology, migration, proliferation, cytokine secretion, and phagocytic activity and can be either beneficial or harmful to the retina [1, 10, 53]. Upon activation, microglia change from their ramified resting state, where they have long and thin processes and a small amount of perinuclear cytoplasm, to an amoeboid hypertrophied state with larger cell bodies and thicker and shorter processes (Fig. 4). Consistently, these characteristic morphological changes of microglial activation were found by immunofluorescence microscopy in retinas of mice subjected to the OIR mouse model [10, 32]. In addition, after activation, retinal microglia cells gain a considerable capacity to migrate and proliferate, an effect that too could be observed in OIR retinas, especially in areas exhibiting ischemia and neovascularization [10]. In contrast to earlier studies in the OIR model, which showed a low proliferation rate of microglia cells [27], recent data demonstrate that microglia do undergo cell expansion and upregulation of essential microglia proliferation markers (e.g., IGF-1, Mif, and Cdk1) at pathological tufts [10]. However, Boeck et al. showed in their study that only every fifth retinal microglia cell had undergone division by postnatal day 17 in the OIR model at the peak of neovascularization. Thus, proliferation alone is insufficient to explain the large increases in microglia cell numbers detectable in the neovascular zones of OIR-treated mice. Rather, it seems more plausible that this accumulation occurs predominantly due to the enhanced migration capacity of microglial cells, another characteristic these cells acquire upon activation [10].

Despite these data, there is still debate about the specific identity of neovascular tuft-associated MPs, because distinguishing microglia from infiltrated MP is often challenging due to the paucity of reliable markers [10]. This is justified in part by a unique expression of typical MP markers including CD11b, CD11c, CD45, CD68, F4/80, and isolectin [53]. However, fate-mapping techniques coupled with flow cytometry revealed that retinal microglia have a uniquely low expression of CD45, CD11c, and F4/80, while monocyte-derived MPs express them in higher quantities. Although, recently, some microglia-specific markers have been identified, e.g., the transmembrane protein 119 and the purinergic receptor P2ry12, under pathological conditions microglia, can be reprogrammed resulting in an upregulation or downregulation of these markers [82]. In addition, it was reported that fluorescently labelled bone marrow cells transplanted into OIR mice migrate to the retina and differentiate into Iba1-positive microglia [51]. This finding further complicates the discrimination between infiltrating MPs and resident microglia, as the two cell types are seemingly able to trans-differentiate into each other. Thus, to date, it is not possible to reliably state which of the MP populations (microglia/blood-derived MP and their sub-populations) is the dominant retinal immune cell in IR. Nevertheless, the sheer increase in MP numbers in the retinal tissue found in the OIR model and in DR [3, 122] makes these cells an interesting target for further studies, irrespective of their specific source.

## Impact of activated retinal mononuclear phagocytes on neovascularization in ischemic retinopathies

### Influence of mononuclear phagocyte number and polarization status

The elevation of MP numbers during the course of the mouse OIR model [10, 24, 27, 51, 74] and in patients with retinopathies [122] raises the question whether these cells are causally involved in the manifestation of IR in a substantial way. Several studies hint that this may be the case, because depletion of MPs in the retina reduces pathological neovascularization in the mouse OIR model [46, 51, 126]. Along these lines, genetic depletion of myeloid mononuclear cells also diminished pathological neovascularization in the same model [60]. In addition, a study performed on OIR mice with a genetic deletion of developmental endothelial locus-1 (Del-1) demonstrated that loss of this leukocyte-repelling gene increased the number of CD45-positive cells and pathological neovascularization in retina [55]. Furthermore, enhanced neovascularization and increased retinal leukocyte infiltration were mechanistically linked, as the increase in neovascularization in Del-1-deficient mice was reversible by blocking leukocyte-endothelium firm adhesion, a prerequisite of leukocyte transmigration, via LFA-1 blockade. Together, these data indicate a correlation between the number of myeloid cells in the retina and the degree of pathological neovascularization. Thus, influencing MP infiltration or proliferation may have therapeutic potential in the treatment of IR.

When assessing the actions of MPs, one has to consider that these cells can shift into different states of activation. Microglia and other MPs generally have the ability to detect and integrate extracellular signals and, depending on these, create different phenotypes, a process termed polarization. So far, at least two distinct phenotypes can be discriminated: the pro-inflammatory M1 and the anti-inflammatory M2 type. MPs of the M1 phenotype are induced by components of a pro-inflammatory microenvironment such as lipopolysaccharide (LPS) or interferon (IFN)-γ and in turn enhance inflammation via the secretion of pro-inflammatory cytokines, including TNFα, interleukin (IL)-1β, IL-6, IL-8, IL-12, and IL-23. In addition, M1-type cells express inducible NO synthase, whereas their secreted levels of IL-10 are low. In contrast, MPs of the M2 phenotype (also known as alternatively activated or immunosuppressive macrophages) are induced by IL-4, IL-10, and IL-13 and enhance gene expression of IL-10 (and arginase-1), while reducing production of IL-6, IL-12, IL-23, and TNFα. Furthermore, M2 cells promote clearance of debris, tissue remodelling, and repair to restore homeostasis [1, 53, 68, 127].

Due to the limited availability of human IR tissue samples, data about MP polarization in the retina and the relevance of this phenomenon for pathological neovascularization in DR and ROP are unfortunately sparse. A study with PDR patients reports that a marker for M2 macrophages (CD163) was overexpressed in the vitreous and in fibrovascular membranes, while an M1 marker (CD80) was below the level of detection in the same samples [56]. In line with this, another study showed that M2 macrophage-related proteins M-CSF and IL-13 were found in elevated levels in the vitreous of patients with PDR [119]. Therefore, M2-polarized MPs may be of importance in the later stages of PDR. In immunostainings of retrolental fibrous membranes of advanced ROP patients, a mixed population of both of M1- (CD40) and M2-MPs (CD206) (with M1-MPs outnumbering M2-MPs [76]) was found, indicating that MPs of both polarization types exist in retinas of patients with the disease.

While these data are merely indicative of the presence of polarized MP subtypes in the retina of patients suffering from IRs, animal models provide a clearer picture of the possible function of polarized MPs in this context. Marchetti and colleagues in vitro differentiated umbilical cord blood-derived human cells into M1 and M2 macrophages and injected them into the vitreous of P7 mice in the OIR model. As a result, reduced pathological neovascularization was obtained with the injection of M2-polarized cells, while injection of M1 cells had no effect [77]. In contrast, in the same mouse model, mouse bone marrow-derived M2 macrophages injected intravitreally at P12 resulted in enhanced pathological neovascularization, while M1 macrophages decreased tuft formation [126]. While the discrepancy between these findings could be related to the differing origin of the macrophages in the two studies, it may also reflect diverging effects of M1-/M2-MPs at different time points of the OIR model. This notion is supported by several studies in the OIR model that assessed time-dependent distribution of M1- and M2-polarized MP in the retina. In this regard, Zhou and colleagues demonstrated that the number of endogenous M2-MPs increased significantly at P17, the time point when the maximum of pathological neovascularization is visible and the transition to the resolution phase is initiated [126]. This is in line with data from Li et al., who also show that M2-polarized MP activity takes over at P17 and peaks at P20, while M1-MP activation starts at P12 (return of the mice to normoxia) and peaks at P17 [68]. Taken together, these data demonstrate that in the OIR model M1 polarization takes place during the phase of pathological neovascularization and transitions into M2 polarization in the later phase of neovascular tuft regression. In light of these findings, it is tempting to speculate that M1-MP may be drivers of neovascularization, whereas M2-MPs may promote resolution of aberrant vasculature. However, this concept has to be tested and developed further in future studies.

The existing evidence strongly suggests that retinal inflammation, including the activation of retinal MPs, might be an important parameter in the progression of IRs. While the exact underlying mechanisms are still unclear, different concepts of how immune cells can manipulate pathological and/or developmental angiogenesis in a direct or indirect manner currently exist. A selection of these theories is addressed in the following.

### Regulation of neovascularization through factors secreted by activated mononuclear phagocytes

Activated MPs alter their expression profile to produce a diverse pattern of cytokines, chemokines, and growth factors, all of which can potentially influence angiogenesis through the modulation of endothelial cell functions such as migration, proliferation, and survival [4].

Interestingly, a variety of these mediators are differentially regulated in patients with PDR and ROP [1, 20, 42, 53, 75, 86, 92, 97, 99, 102, 113]. A selection of important secreted mediator molecules and how they are altered in the aforementioned pathologies is depicted in Table 1.

**Table 1:** Regulation of secreted mediators (e.g., cytokines, chemokines, and growth factors) in ROP and DR

Name	Regulation in ROP	Regulation in DR
VEGF	↑	↑
Epo	↑	↑
Opticin	↓	Nd
IGF-1	↓	↑
TNFα	↑	↑
IL-1β	Nd	↑
IL-1α	Nd	↑
IL-4	Nd	↑
IL-6	↑	↑
IL-8	↑	↑
IL-10	↑	↑
IFNγ	↑	↑
MCP-1 (CCL-2)	↑	↑
MIP-1β (CCL-4)	↑	↑
CX3CL1 (Fraktalkine)	Nd	↑
MMP2/9	↑	↑

Arrows indicate upregulation (up arrow) or downregulated (down arrow) of the mediators in eyes with the respective pathology as compared to eyes free of retinopathy. References [1, 20, 42, 53, 75, 86, 92, 97, 99, 102, 113]

*Nd* levels not defined, *VEGF* vascular endothelial growth factor, *EPO* erythropoietin, *IGF-1* insulin-like growth factor 1, *TNFα* tumor necrosis factor α, *IL* interleukin, *IFNγ* interferon γ, *MCP-1* monocyte chemoattractant protein 1, *MIP-1β* macrophage inflammatory protein-1β, *MMP2/9* matrix metalloproteinase-2/9

Following, several important secreted factors and their potential effects on retinal neovascularization are described. While these factors can be produced and secreted by MPs, it should be noted that they can also be derived from other retinal cell types (e.g., Muller cells and neurons), especially following injury.

#### VEGF and opticin

VEGF, a well-studied and highly potent pro-angiogenic molecule, has already been identified as one of the main drivers of pathological angiogenesis in PRs, and it is therefore targeted in the pharmacological anti-VEGF treatments as described above. However, VEGF produced by retinal MPs is unlikely to be a major contributor to the high retinal levels of VEGF in patients with PRs, because compared to other retinal cell types, the number of MPs in this tissue is small, even during retinopathies. This is confirmed by previous findings [74], which indicate that retinal microglia are not a major source of VEGF in the ischemic retina and are thereby unlikely to contribute to development of IR through this mechanism [10]. Although, it is possible that other MP-derived mediators modulate angiogenesis in a secondary fashion by acting on non-endothelial cell types that in turn produce pro-angiogenic factors. For example, a recent study shows that under hypoxic conditions, macrophages can activate Mueller cells, which then in turn express VEGF [81].

While the pro-angiogenic effects of VEGF are researched in many scenarios, the inflammatory actions of this mediator are often overlooked. In this regard, Ishida and colleagues found that a subform of VEGF (VEGF_164_) drives pathological, but not physiological angiogenesis in OIR rats, by attracting MPs to the site of neovascular tuft formation [46]. Additionally, other works reported that VEGF has the ability to directly activate MP migration and cytokine production through binding of the VEGF receptor 1 on these cells [6, 80]. Therefore, it is tempting to hypothesize that the high VEGF levels in the retina during PRs may contribute to MP attraction and activation in these settings. VEGF in the retina may therefore drive pathological angiogenesis not only directly through angiogenic signaling, but potentially also indirectly through the promotion of retinal inflammation.

Even though MPs are not a major source of VEGF, they may still regulate pathological retina angiogenesis through other molecules. Opticin is an anti-angiogenic molecule secreted by microglia cells. It counters angiogenesis by weakening the adhesions of endothelial cells to collagen and thereby prevents pro-angiogenic integrin signaling [63]. Interestingly, opticin is downregulated in microglia under hypoxia and, consistently, decreased opticin levels were observed in the vitreous from ROP patients [86]. Retinal opticin levels seemingly have a strong influence on pathological retinal angiogenesis, as opticin-deficient mice showed elevated OIR neovascularization [62] and intravitreal injection of recombinant opticin protected against pathological retina angiogenesis in the same model [54]. Therefore, alterations to secreted opticin amounts by retinal MPs may be a major factor determining the extent of pathological angiogenesis in this tissue.

#### Matrix metalloproteases

Another class of immune cell-derived molecules that might regulate neovascularization are matrix metalloproteases (MMPs). MMPs are secreted proteases that can break down and remodel the extracellular matrix (ECM) associated with the endothelium. This may lead to the mobilization of proangiogenic growth factors that would otherwise remain inaccessibly embedded within the perivascular matrix [4, 115]. MMP levels, especially of MMP-2 and MMP-9, are indeed enhanced in the retina of diabetic and ROP patients [59, 86, 92] as well as in animal models of diabetes [1, 28]. Furthermore, pharmacologic and genetic inhibition of MMP-2 and MMP-9 reduced pathological retinal neovascularization in the OIR model [7]. In line, MMP-9 derived from microglia cells was able to degrade the aforementioned opticin, hinting at a potential additional mechanism by which MMPs may influence angiogenic processes [86]. This highlights the functional relevance of MMP activation for pathological neovascularization in the retina.

#### Cytokines (TNFα, IL-1β, INFγ, MCP-1)

Activated MPs typically secrete a wide array of inflammatory cytokines and chemokines depending on their polarization status. These cytokines can further enhance activation of MPs, which then in turn secrete chemokines to recruit additional MPs to the sites of inflammation, potentially causing an amplification of the inflammatory process. It is noteworthy that several studies report increased presence of several inflammation-associated cytokines in eyes of patients with retinopathies, which raises the question whether these cytokines are indicators and/or drivers of IR manifestation. Specifically, diabetic patients with DR regularly exhibit enhanced vitreal levels of TNFα, Il-1β, Il-6, IL-8, INFγ, and MCP-1 [100, 113, 120]. Similarly, in ROP patients, a study of Lyu et al. shows that protein levels of TNFα, Il-6, IL-8, and INFγ were increased in eyes from infants with ROP pathology in comparison to those from infants without ROP [75, 92]. In addition to these correlative studies, data from animal models suggest causal links between inflammatory cytokines in the retina and IR-associated pathological angiogenesis. In this regard, two studies on TNFα-deficient mice demonstrated that the lack of this cytokine enhances the number of retinal microglia cells. In parallel, OIR-induced pathological neovascularization was reduced, physiological revascularization improved, and oxidative stress as well as retinal cell apoptosis decreased [33, 107]. Another work demonstrated that the predominant source of TNFα in the OIR retina is MPs. Moreover, it found indications that this TNFα stimulates retinal glia cells to in turn produce the angiogenic and inflammatory factors basic fibroblast growth factor, IL-8, and MCP-1 [118]. In agreement with these findings, attenuation of retinal neovascularization in the OIR model was achieved by pharmacological inhibition of TNFα activity via the TNFα receptor antagonist Etanercept or the anti-inflammatory nutrient ω-3-PUFA, opening potential opportunities for new treatment options [22, 117]. IL-1β, another well-studied pro-inflammatory cytokine, is produced early in OIR (earlier than TNFα) by retinal microglia cells and promotes early vaso-obliteration via induction of endothelial cell apoptosis [93], an effect that likely contributes to pathological neovascularization in later stages. Together, these data strongly suggest that pro-inflammatory cytokines, such as TNFα and IL-1β produced by retinal MPs, drive cellular stress and pathological angiogenesis in the retina.

However, not all cytokines necessarily promote retinal neovascularization. One report by Jung et al. found that IFNγ, another well-known pro-inflammatory cytokine, surprisingly exhibited potent anti-angiogenic efficacy in the mouse model of OIR, likely by suppressing VEGF-induced angiogenesis in endothelial cells [49]. Furthermore, MCP-1, a chemokine that facilitates transmigration of MPs across endothelial monolayers [29] and whose production is increased by activated microglia in OIR [10], exerted a surprising effect in the OIR model. When mice lacked MCP-1, they formed the same amount of retinal neovascular tufts, but these structures exhibited a reduced number of recruited MPs and apoptotic cells, which ultimately led to a delay in neovascular tuft regression [25]. These data suggest that MCP-1 is not directly involved in pathological neovascularization, but may promote tuft resolution by attraction of retinal MPs.

Taken together, there are several indications that inflammatory cytokines could modulate pathological retina angiogenesis, but the underlying mechanisms are incompletely understood to date.

#### Nerve growth factor

Another class of secreted modulators that are expressed in the retina are neurotrophins, which were shown to be elevated in DR patients [11, 97]. Especially, nerve growth factor (NGF) levels were higher in the vitreous, in the serum, and in the tears of patients with DR [11, 85]. In line, NGF levels in retinas of pups subjected to the OIR model displayed an increased NGF expression [72] and topical application of additional NGF further enhanced, while intraocular injections of anti‐NGF neutralizing antibody reduced pathological retinal vascularization in mice subjected to the OIR model [112]. The elevated NGF levels observed in IR might be a result of the enhanced neurodegeneration and inflammation. Inflammatory cytokines, such as TNFα, IL-1β, and IL6, are promotors of NGF synthesis in a variety of retinal cell types, e.g., in ganglion cells, bipolar cells, glial cells, retinal pigment epithelial cells, and MPs [79]. Little is known about the exact mechanism of NGF-mediated angiogenic effects. NGF can act on several cells, which express NGF receptors, including endothelial cells and MPs. The pro‐angiogenic effect of NGF on endothelial cells was mediated by the inhibition of retinal endothelial cell apoptosis [112]. In addition, binding of NGF on MPs may stimulate pro-inflammatory cytokine production (TNFα and IL-8), which could further boost inflammation in IR [43]. Taken together, several studies point to a potential contribution of the NGF-microglia axis to the development of IR; however, further investigations are needed.

#### Insulin-like growth factor 1

As mentioned before, polarization of MPs is an important factor that determines the specific form of activity and the pattern of secreted factors in these cells. In this context, insulin-like growth factor 1 (IGF-1), which is also secreted by MPs, may play a special role in the manifestation of IR. A study performed in bone marrow-derived mononuclear cells revealed that IGF-1 drives IL-4-mediated M2 polarization and counters IFNy-mediated M1 polarization, pointing to proangiogenic/tissue repair as well as anti-inflammatory properties [8, 110]. It is not yet fully clear whether IGF-1 exhibits these properties in the retina as well. However, a work studying intracerebral hemorrhage found signs that injection of recombinant IGF-1 had positive therapeutic and anti-inflammatory effects, which were mechanistically linked to M2 polarization of the brain microglia [108]. Hence, IGF-1 could have similar effects on retinal MPs, and given the link between inflammation and IR manifestation, IGF-1 may therefore potentially counter IR manifestation by promoting M2 polarization. In fact, a curative effect of IGF-1 was demonstrated in the OIR model, where intraocular injection of an IGF-1 overexpression construct attenuated retinal neovascularization and blood-retina barrier breakdown, while injection of an IGF-1-siRNA construct had the opposite effect [66]. In addition, low levels of blood IGF-1 in prematurely born infants have been linked to an increased risk of ROP manifestation later on in these children, which strengthens the potential role of IGF-1 in this disease [42, 71]. Similar studies in the field of DR are currently not available. Yet, a work by Burgos et al. depicted upregulation of IGF-1 levels in the vitreous of patients with proliferative DR, hinting that IGF-1 could play a role in this form of IR as well [15]. Therefore, it is tempting to hypothesize a general causal relationship between IGF-1 concentrations in the retina and manifestation of IR, which may be linked to the M2-polarizing effects of IGF-1.

Taken together, diverse factors produced and secreted by MPs may have the potential to influence the progression and manifestation of IRs.

### Regulation of neovascularization through cell–cell interactions between mononuclear phagocytes and endothelial cells

The pro-inflammatory microenvironment in retinas during the manifestation of IR leads to the upregulation of adhesion molecules such as ICAM-1, VCAM-1, and E-selectin on the apical surface of the endothelium [52, 78, 102]. These molecules facilitate the firm binding of MPs and other leukocytes to the endothelium and thereby bring these cell types in prolonged direct contact to each other. This process has been observed especially in areas of neovascularization and raises the question whether MPs can influence endothelial cells directly via cell–cell-based interactions [24, 27]. According to a recent study by Liu et al., endothelial cells and MPs that are directly associated with each other in neovascular tufts and there mutually influence one another via the exchange of glycolytic metabolites (likely lactate). Supposedly, this interaction causes the neighboring MPs to acquire a mixed polarization state in which they simultaneously produce pro-angiogenic and pro-inflammatory mediators [73].

MPs might also kill endothelial cells they are in direct contact with. This process likely takes place in neovascular tufts formed in the OIR model, as they often contain apoptotic endothelial cells [25, 57]. There, MPs are reported to drive endothelial cells into BRB breakdown and apoptosis via ROS production and binding of their membrane-associated apoptosis inducer Fas ligand (FasL) to the endothelial Fas [40, 47]. In fact, this process has been demonstrated in a study using mice with hematopoietic Hif2α deficiency, which caused downregulation of a disintegrin and metalloprotease 17 in MPs and thereby increased expression of FasL in cells of the hematopoietic linage, including MPs. This effect led to increased endothelial cell apoptosis in pathological vessels and resulted in reduced neovascularization in the OIR model. Hence, MPs in pathological tufts exhibited an increased potential to induce endothelial cell apoptosis via direct cell–cell contact and thus could attenuate neovascularization [57]. Moreover, according to Dehn et al., Hif2α in immune cells can act as a phagocytosis repressor [26]. Therefore, Hif2α-deficient mice may have reduced pathological angiogenesis additionally due to more efficient clearance of apoptotic/dying endothelial cells by MPs with enhanced phagocytic capabilities. The process of apoptotic/dead cell removal by phagocytosis is termed efferocytosis and is required to prevent the exposure of the surrounding tissue to uncontrolled enzyme activity. Efferocytosis is described in many inflammatory conditions [30], but studies about its role in scenarios of pathological retinal angiogenesis are lacking at this point in time. In ROP and the OIR model, pathological tufts are typically cleared after a while, resulting in the normalization of the retinal vessels and subsequent healing. As this phenomenon necessitates a tremendous reduction in endothelial cell numbers within the retina, it seems plausible that effective MP-mediated phagocytosis/efferocytosis is required to remove these endothelial cells and their remnants. Therefore, it seems tempting to assume that phagocytic activity within the retinal tissue may be an important parameter that counters neovascularization. Indications of phagocytic activity of MPs within the retinal tissue were discovered by Poche et al., who observed that MPs in the retina are able to engulf endothelial cell membrane particles in the physiological process of pupillary membrane capillary regression [89]. In addition, there are indications that phagocytosis by MPs may regulate angiogenesis [58]. In an in vitro model that assessed phagocytic activity of cultured MPs (microglia and BMDM) towards apoptotic endothelial cells, it was shown that genetic deficiency of myeloid suppressor of cytokine signalling 3 (SOCS3) in MPs increased their phagocytic rates and that this effect was mediated by enhanced expression of growth arrest-specific gene 6 (Gas6), an important phagocytosis inducer [35, 58]. Interestingly, myeloid SOCS3 deficiency reduced sprouting activity of endothelial cells in an ex vivo aortic ring model, and this effect could be reversed through simultaneous blockage of the Gas6 receptor Mer [58].

The previously mentioned studies suggest that efferocytosis is a protective mechanism of MPs in IR that attempts to clear neovascularization in order to prevent the formation of pathological vessels or resolves existing neovessels. However, it is conceivable that efferocytosis could have adverse effects in the early stages of IR. This could be the case in DR, if removal of stressed cells in existing vessels by MPs would destabilize these vessels. Very few studies have addressed this hypothesis, but a recent report suggested that perivascular MPs remove pericytes through efferocytosis in eyes of diabetic patients [34]. It is not clear, however, whether this phenomenon is harmful or beneficial to retina vessel integrity in diabetics. Therefore, pericyte efferocytosis may as well be another protective effect of MPs that limits local inflammation induction via removal of dead cells remnants.

Taken together, these data indicate that efferocytosis by MPs might play an additional role in the regulation of angiogenesis.

## Conclusion

Ischemic retinopathies remain a significant medical problem and are among the major causes of blindness in the western world. While efficient and widely available, the current treatment options do not always prevent the progression of IR and can have unwanted side effects. Thus, there is still a high demand for the development of novel intervention strategies that are based on different aspects of pathophysiology and new molecular and cellular mechanisms.

Recent studies show that the retinal tissue is exposed to enhanced cellular stress and subsequent inflammation during the manifestation and progression of IRs. These inflammatory processes trigger activation and infiltration of MPs, which can in turn promote and sustain inflammation. Activated MPs can lead to tissue destruction, but may also promote tissue regeneration and resolution of the disease. This diametrically opposed behavior of MPs may be explained by their ability to polarize into different states, wherein M1 polarization mainly causes tissue destruction and inflammation and M2 polarization mediates tissue regeneration and counters inflammation. However, the influence of these MP polarization states on IR development is not straight forward. Activated MPs can impact pathological retina angiogenesis through a wide array of actions. They have the potential to drive neovascularization through the secretion of matrix metalloproteases (MMP-2, MMP-9) or pro-inflammatory cytokines (TNFα, IL-1β, and IL-6) that extend inflammation and promote leukocyte-endothelial adhesion. MPs may also counter pathological angiogenensis via secretion of pro- and anti-angiogenic factors, and by killing (FasL-Fas interaction) and phagocyting stressed/apoptotic endothelial cells.

Taken together, inflammatory processes contribute to the development of IRs both in early and late stages. Retinal cell stress and inflammation triggers activation and recruitment of MPs that can both drive and counter disease progression. Therefore, retinal MPs represent a promising target for the development of novel intervention strategies against IR, but the knowledge about the regulatory and effector mechanisms of inflammation in the retina must first be expanded in order to achieve this goal.

## Data Availability

Not applicable; no supporting data.
